# Endovascular treatment of symptomatic severe intracranial atherosclerotic stenosis with a novel intracranial dedicated drug-eluting stent: a more effective treatment approach

**DOI:** 10.3389/fneur.2024.1304524

**Published:** 2024-02-22

**Authors:** Lin Ma, Fei Wang, Hao Feng, Shuo Yan, Ji-Chong Xu, Ying-Sheng Cheng, Chun Fang

**Affiliations:** ^1^Department of Interventional Radiology, Tongji Hospital, School of Medicine, Tongji University, Shanghai, China; ^2^Department of Neurosurgery, Tongji Hospital, School of Medicine, Tongji University, Shanghai, China

**Keywords:** intracranial stenosis, angioplasty, stenting, drug-eluting stent, in-stent restenosis

## Abstract

**Background:**

Endovascular treatment of severe intracranial atherosclerotic stenosis (ICAS) using coronary drug-eluting stents (DESs) significantly reduces the risk of in-stent restenosis (ISR) and stroke recurrence. However, there are few reports regarding the treatment of ICAS with intracranial dedicated DES. Herein, we present our experience with the feasibility, safety, and medium-term follow-up outcomes of a novel intracranial DES, named NOVA stent, in patients with symptomatic severe ICAS (≥70%).

**Methods:**

From December 2021 to May 2022, patients with symptomatic severe ICAS who underwent implantation of the NOVA stent in our institution were retrospectively analyzed for procedural results, perioperative complications, imaging and clinical follow-up outcomes.

**Results:**

Twenty-four patients, 16 (66.7%) with anterior circulation lesions and 8 (33.3%) with posterior circulation lesions, were enrolled. All patients with intracranial ICA (*n* = 6), middle cerebral artery (*n* = 10), basilar artery (*n* = 3), intracranial vertebral artery (*n* = 3), and the vertebrobasilar junction (*n* = 2) stenosis were treated successfully using NOVA stents. The severity of stenosis ranged from 75 to 96% (mean 85.9%) before treatment and this was reduced to 0 to 20% (mean 8.6%) immediately after stent placement. Symptomatic distal embolism occurred in one case; however, there were no other perioperative complications. The mean follow-up duration was 12.2 ± 1.06 months. No symptomatic ischemic events occurred during follow-up. Follow-up cerebral angiography was performed in 22 of 24 patients (91.7%), and significant ISR occurred in one patient (4.2%).

**Conclusion:**

Our results demonstrate that implantation of the novel intracranial DES NOVA in severe ICAS is feasible, safe, and effective in selected cases, reducing the incidence of ISR, and showing excellent midterm clinical outcomes, providing a promising option for ICAS treatment.

## Introduction

The incidence of stroke caused by intracranial atherosclerotic stenosis (ICAS) is higher in Asians than in Caucasians (1–3). Although standard medical therapy is still the preferred treatment for stroke prevention in patients ICAS, there is still a certain recurrence rate of stroke in a subset of patients, even after the maximum drug treatment, especially in patients with severe intracranial stenosis (≥70%), thus necessitating the application of stenting in these patients (4–9). However, the high complication rate associated with stenting, and the in-stent restenosis (ISR) rate, limits the application of stents in symptomatic ICAS (10–14). With the improvement of neurointerventional techniques and the development of instruments, periprocedural complications seem to have been satisfactorily controlled. The Wingspan Stent System Post Market Surveillance (WEAVE) trial and the Registry Study of Stenting for Symptomatic Intracranial Artery Stenosis in China showed periprocedural complication rates of 2.6 and 4.3%, respectively, suggesting that intracranial stenting might be safe for use in selected patients with ICAS (15–18). However, ISR caused by neointimal hyperplasia after stent implantation is reported to be as high as 33%, a major problem that requires an urgent solution (13, 14). The invention and application of drug-eluting stents (DESs) in the coronary artery has been shown to significantly reduce the incidence of ISR (19, 20). The DES is designed to reduce ISR after stent implantation by inhibiting the migration and proliferation of vascular endothelial cells and smooth muscle cells (21, 22). In some previous studies, the use of coronary DESs to treat intracranial stenosis showed good feasibility and safety, and efficacious clinical outcomes. Compared with conventional bare-metal stents, the coronary DESs used for intracranial stenosis showed a lower rate of both ISR and stroke recurrence (23–26). However, there are few reports regarding the treatment of ICAS with DESs designed specifically for the intracranial artery. In 2022, Miao et al. reported the results of applying a novel intracranial DES NOVA in patients with symptomatic severe ICAS for the first time (27). NOVA stent is the world’s first balloon-expandable DES specifically designed to treat ICAS, and is also the world’s first healing-oriented intracranial stent. The NOVA stent has a coating thickness of 3.8 to 10 μm, is composed of a poly (D,L-lactide-co-glycolide) biodegradable polymer, and is coated with sirolimus at a density of 1.3 μg/mm^2^. The technique used to produce the NOVA stent is the patented eG^™^ electron grafting coating technology. The unique drug-coating design enables rapid endothelial coverage and repair within the endothelial repair window, reducing the incidence of ISR and the risk of ischemic events recurrence. However, there is insufficient experience on NOVA stent application. This study reports the single-center clinical experience of the NOVA stent to treat symptomatic severe ICAS after its launch, with the aim of determining its safety, technical feasibility, and the medium-term ISR rate after NOVA placement in patients with ICAS lesions, to provide more experience for the clinical application of intracranial DES.

## Methods

### Patient selection

From December 2021 to May 2022, patients with symptomatic severe ICAS who underwent implantation of the NOVA stent in our institution were retrospectively analyzed for procedural results, perioperative complications, imaging and clinical follow-up outcomes. We performed DES placement in patients if they met the following criteria: (1) Severe intracranial atherosclerotic stenosis radiologically reduction (≥70%), confirmed by digital subtraction angiography (DSA) following Warfarin-Aspirin Symptomatic Intracranial Disease Study (WASID) criteria (28); (2) ischemic stroke caused by the intracranial atherosclerotic stenosis; (3) head non-contrast computed tomography (NCCT) or magnetic resonance imaging (MRI) showing cerebral watershed infarction, and CT or MRI perfusion showing hypoperfusion on responsible lesion; (4) high resolution magnetic resonance imaging (HR-MRI) revealing unstable plaques; and (5) continuing, aggravated, or recurrent ischemic neurological deficits despite maximal medical therapy. Patients were not conducted when the following conditions were present: (1) severe hemorrhagic transformation of the infarction zone; (2) severe angulation of the stenosis vessel (>45°) or excessive distortion in the proximal segment making it unsuitable for balloon-expandable stents.

### Preoperative preparation

All patients received with daily doses of 100 mg of aspirin and 75 mg of clopidogrel for ≥5 days before stenting. A thrombelastogram (TEG) test was then performed to rule out drug resistance. For patients resistant to clopidogrel, we changed the drug to ticagrelor (90 mg bid). Cholesterol, blood sugar, blood pressure, and other vascular risk factors of the patients were satisfactorily controlled. A thorough cerebral angiography should be performed for each patient to comprehensively evaluate the anatomical characteristics of the lesion vessel, including the lesion length, vessel diameter, and the position of perforating arteries or branch vessels. NCCT was conducted 1 day before intervention to rule out hemorrhage transformation in the cerebral infarction area for patients with stroke. All the patients underwent evaluation by a neurologist before the revascularization procedure.

### Procedures for NOVA placement

All procedures were performed under general anesthesia by experienced neurointerventionalists using a standardized process created by our center. Heparin (70 U/kg) was administered intravenously to achieve an activated clotting time between 250 and 300 s. A 6-French shuttle sheath (Cook, Bloomington, IN, United States) was positioned in the distal common carotid artery or subclavian artery close to the vertebral artery orifice. A 5-French intermediate catheter was placed at the proximal end of the stenosis through the shuttle sheath. Under road map fluoroscopy, a 205 cm-long and 0.014-inch-diameter microguidewire (Transcend, Boston Scientific, San Jose, CA, United States) was navigated through the stenotic vessel. According to the operator’s judgment, predilation of the stenotic segment was performed using a rapid exchange intracranial balloon dilation catheter (Neuro LPS, SINOMED, Beijing, China). The balloon size was selected according to the diameter of the normal vascular segment near the lesion. The maximum diameter of the balloon selected was less than 80% of the diameter of the lesion, and the length of the balloon was as short as possible on the basis of covering the lesion length. The selected DES stent (NOVA Intracranial Sirolimus Eluting Stent System, SINOMED) was then propelled through the satisfactory dilated lesion vessel and implanted by inflating the balloon to the prescribed pressure. The stent diameter was selected to be slightly smaller than the adjacent normal artery diameter, and the stent length was selected to cover the end of both sides of the stenotic segment by 3 mm. To avoid vessel dissection and rupture, a slow inflation needed to be performed ranging from 15 to 30 s per atmosphere at the time of angioplasty or stent deployment. The balloon was needed to be deflated slowly while the patient’s systolic blood pressure (SBP) was needed to be controlled below 120 mmHg or 20 mmHg below the basic SBP to prevent hyperperfusion syndrome (HPS). Residual stenosis, perforator vessel occlusion and distal perfusion were evaluated by postoperative angiography. A reduction in stenosis to ≤30% of the luminal stenosis and the occurrence of no vessel dissection or visible branch occlusion is considered a technical success. Periprocedural complications, such as transient ischemic attack (TIA), hemorrhage, minor stroke [modified Rankin Score (mRS) ≤ 2], or major stroke (mRS > 2) were evaluated after stenting.

### Postprocedural management

After surgery, all patients were transferred to the neurological intensive care unit for observation and continuous arterial blood pressure monitoring to keep the SBP between 100 and 120 mmHg. A brain CT was immediately executed to exclude post-procedure hemorrhage. After surgery, if the patient’s symptoms worsen or new symptoms appear, head MRI would be performed to determine whether the cause was caused by distal embolism. Patients continued to take dual antiplatelet drugs for at least 6 months after the procedure, and then decide whether to suspend one drug based on the results of follow-up imaging.

### Imaging and clinical follow-up

Imaging follow-up was performed at 6 months and 12 months after the operation using head CTA or DSA, and then annually thereafter. In-stent restenosis (ISR) was measured according to the WASID criteria (28). A radiologically reduction >50% within the stent or just outside the stent margins was confirmed as significant ISR (29). Clinical follow-up was performed at the outpatient clinic or at each admission with imaging follow-up. Any neurological deficits differing from baseline neurological status that were related to the procedure were assessed and recorded. Patients with any deterioration in neurological status were required to return to the hospital and undergo a head MRI if necessary during the follow-up period. Patients’ clinical state were evaluate by mRS score. Data on the initial and final clinical outcomes and angiographic results were collected and analyzed by two experienced neurointerventionalists.

### Statistical analysis

The SPSS software package, version 16.0 (IBM Corp., Armonk, NY, United States), was used in this study for statistical analysis. For continuous variables, data are presented as the mean ± standard deviation; for continuous variables with skewed distributions, data are presented as the median; and for nominal variables, data are presented as percentages.

## Results

### Baseline characteristics of the patients

A total of 24 patients (18 male and 6 female) were enrolled, with an age range of 45 to 76 years (median age, 62 years). The median time between the qualifying event and DES implantation was 24 days (range, 8–56 days). All patients had a history of TIA and/or stroke. Fifteen patients had recurrent ischemic neurological deficits (62.5%), four patients had progressive neurological impairment (16.7%), and five had recurrent TIA attacks (20.8%). The stenoses were located as follows: intracranial internal carotid artery (ICA; *n* = 6, 25%), middle cerebral artery (*n* = 10, 41.7%), basilar artery (*n* = 3, 12.5%), intracranial vertebral artery (*n* = 3, 12.5%), and the vertebrobasilar junction (*n* = 2, 8.3%). Risk factors for ischemic events included hypertension (*n* = 21, 87.5%), diabetes mellitus (*n* = 10, 41.7%), hyperlipidemia (*n* = 18, 75.5%), a history of smoking (*n* = 14, 58.3%), and coronary artery disease (*n* = 8, 33.3%). The mean mRS score was 1.67 ± 1.09 before stenting, including a score of 0 in five cases, a score of 1 in four cases, a score of 2 in nine cases, and a score of 3 in six cases. The baseline characteristics of the patients are summarized in Tables 1, 2.

**Table 1:** Baseline characteristics of 24 patients with ICAS.

Characteristic	No. (%)
Sex	
Male	18 (75%)
Female	6 (25%)
Age, median (IQR), y	62 (45–76)
Stenosis location	
Intracranial ICA	6 (25%)
Middle cerebral artery	10 (41.7%)
Basilar artery	3 (12.5%)
Intracranial vertebral artery	3 (12.5%)
Vertebrobasilar junction	2 (8.3%)
Medical history	
Hypertension	21 (87.5%)
Diabetes mellitus	10 (41.7%)
Hyperlipidemia	18 (75.5%)
Smoking history	14 (58.3%)
Coronary artery disease	8 (33.3%)
Qualifying event	
Ischemic stroke	15 (62.5%)
TIA	5 (20.8%)
SIP	4 (16.7%)
Time from qualifying event to the procedure, median (IQR) Days	24 (8–56)
Stenosis of qualifying artery, mean	85.9% (75–95%)
mRS score, mean ± SD	1.67 ± 1.09

IQR, interquartile range; ICA, internal carotid artery; TIA, transient ischemic attack; SIP, stroke in progression; mRS, modified Rankin Scale.

**Table 2:** Baseline demographic and clinical information of the enrolled patients.

Patient no.	Age (yr)/Sex	Clinical presentation	Lesion location	Initial mRS	Pre-stenting stenosis (%)	Post-stenting stenosis (%)	Stent size (mm)	Follow-up				
								Time (months)	Ischemic event	Imaging modality	ISR	Last mRS
1	64/M	Stroke	MCA	2	85	5	2.25 × 12	14	No	DSA	No	1
2	67/F	TIA	ICA	0	90	10	2.75 × 15	12	No	DSA	No	0
3	68/M	Stroke	MCA	3	92	0	2.25 × 12	13	No	DSA	No	2
4	68/M	TIA	IVA	0	88	0	4.0 × 15	12	No	DSA	No	0
5	76/M	Stroke	ICA	3	95	5	4.0 × 12	14	No	DSA	No	2
6	54/M	Stroke	BA	1	76	10	3.0 × 15	12.5	No	DSA	20	0
7	64/F	SIP	ICA	3	90	15	3.5 × 12	13	No	DSA	No	2
8	52/M	Stroke	MCA	1	90	5	2.25 × 10	11	No	CTA	No	0
9	70/M	Stroke	IVA	2	92	12	2.5 × 12	12	No	DSA	No	1
10	54/M	SIP	MCA	3	95	20	2.25 × 12	12.5	No	DSA	No	2
11	64/M	Stroke	ICA	2	85	15	3.0 × 15	13.5	No	DSA	55	1
12	60/M	TIA	MCA	0	78	10	2.25 × 10	11	No	DSA	No	0
13	59/M	Stroke	MCA	2	75	5	2.25 × 12	13.5	No	DSA	No	1
14	45/M	Stroke	BA	2	75	5	3.0 × 15	12	No	CTA	No	1
15	75/F	Stroke	MCA	2	80	8	2.25 × 15	12.5	No	DSA	No	1
16	62/F	TIA	MCA	0	85	5	2.25 × 12	13	No	DSA	20	0
17	62/M	Stroke	IVA	1	75	0	2.5 × 12	10	No	DSA	No	0
18	63/M	Stroke	VBJ	1	78	10	2.25 × 12	11.5	No	DSA	No	0
19	67/M	TIA	MCA	0	88	8	2.25 × 12	12	No	DSA	No	0
20	58/M	SIP	ICA	3	90	15	3.5 × 20	12	No	DSA	No	2
21	53/F	Stroke	ICA	2	90	13	3.5 × 15	10	No	DSA	No	1
22	62/M	Stroke	VBJ	2	90	15	2.5 × 10	11.5	No	DSA	No	1
23	70/F	SIP	MCA	3	95	10	2.25 × 10	12	No	DSA	No	2
24	65/M	Stroke	BA	2	85	5	2.75 × 12	12.5	No	DSA	No	2

TIA, transient ischemic attack; SIP, stroke in progression; ICA, internal carotid artery; MCA, middle cerebral artery; BA, basilar artery; IVA, intracranial vertebral artery; VBJ, vertebrobasilar junction; DSA, digital subtraction angiography; CTA, computed tomography angiography; ISR, in-stent restenosis; mRS, modified Rankin Scale; M, male; F, female.

### Primary procedural results

**Figure 1:**
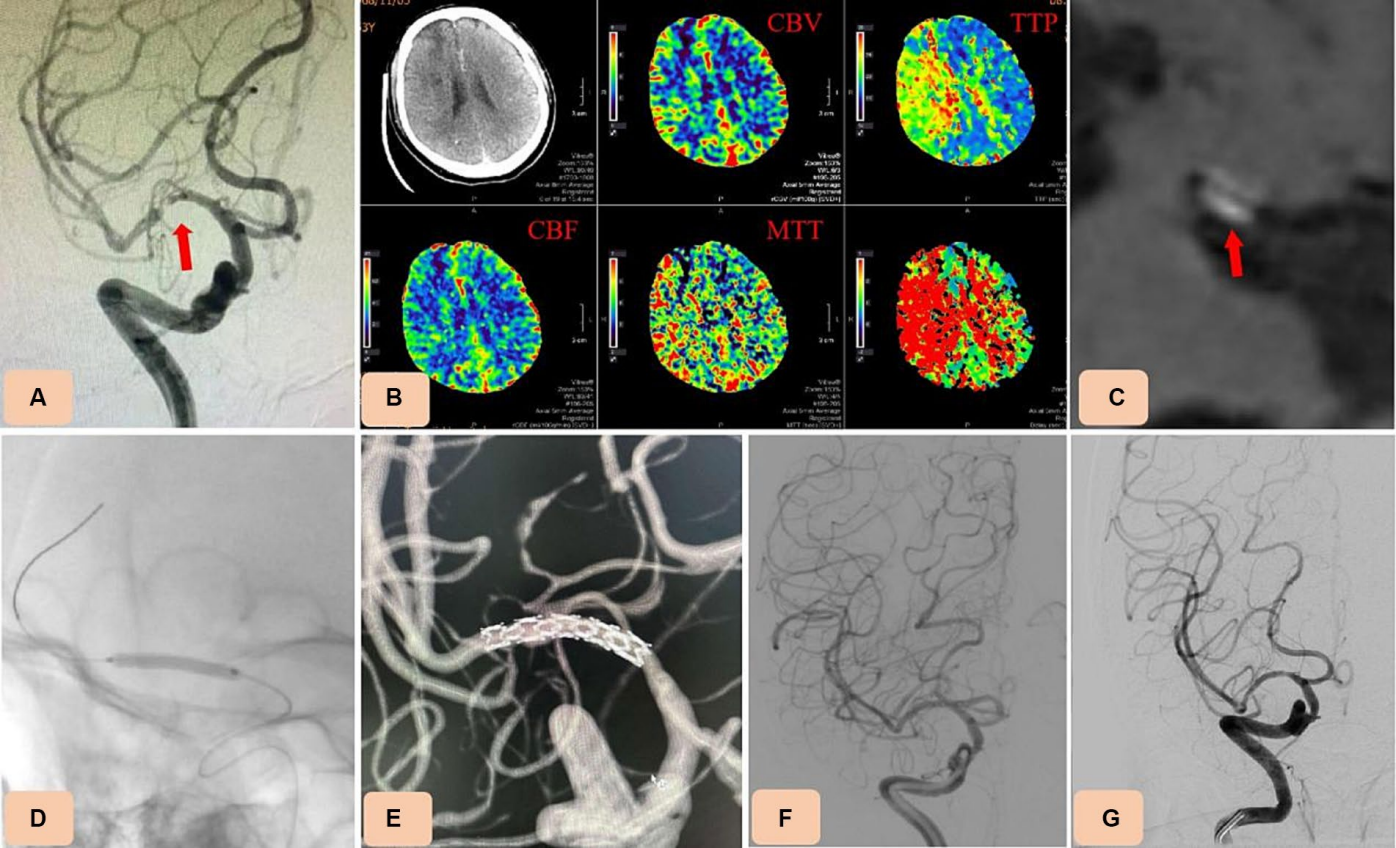
Case 3. Percutaneous transluminal angioplasty and stenting (PTAS) for severe M1 segment stenosis of the right middle cerebral artery (MCA) with the novel drug-eluting stent (DES), NOVA. **(A)** Preprocedural digital subtraction angiography showing severe M1 segment stenosis of the right MCA (arrow). **(B)** Computed tomography perfusion (CTP) showing hypoperfusion on the right MCA territory (increase of Time-to-peak (TTP) and Mean transit time (MTT)). **(C)** High resolution magnetic resonance imaging (HR-MRI) revealing unstable plaques (arrow). **(D,E)** A selected NOVA stent (2.5 mm × 12 mm) was advanced across the stenotic vessel and inserted by inflating the balloon. **(F)** Postoperative angiography confirmed the in-stent patency, with no residual stenosis. **(G)** Thirteen-month follow-up angiography showing no in-stent restenosis.

**Figure 2:**
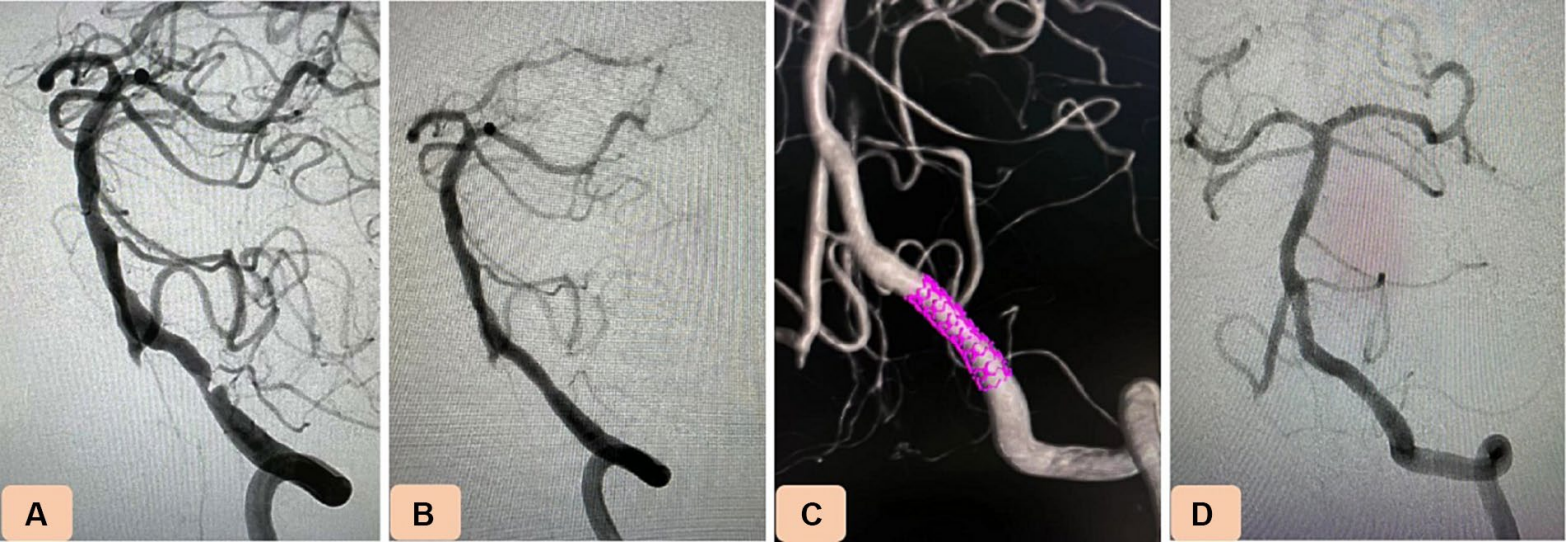
Case 4. Novel drug-eluting stent (DES), NOVA implantation in severe stenosis of the intracranial vertebral artery (VA). **(A)** Pretreatment digital subtraction angiography revealing 88% stenosis of the intracranial segment of the nondominant left VA. **(B,C)** After balloon predilation, a NOVA stent (3.5 mm × 15 mm) was navigated and placed in the stenotic vessel. **(D)** Twelve-month follow-up angiography showing no in-stent restenosis.

**Figure 3:**
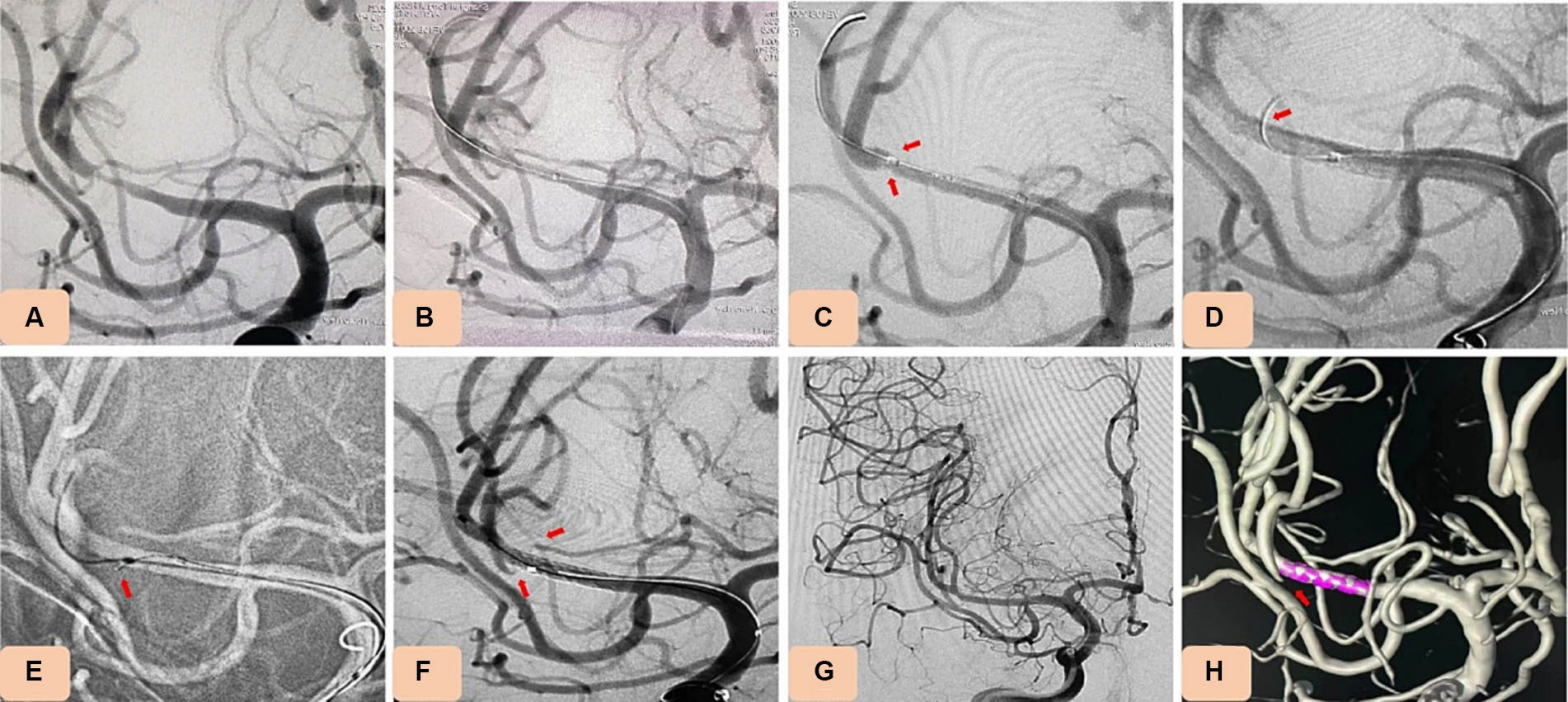
Case 15. Novel drug-eluting stent (DES), NOVA implantation in severe stenosis of M1 segment of the middle cerebral artery (MCA). **(A)** Pretreatment digital subtraction angiography (DSA) showing severe stenosis of M1 segment of the MCA (80% stenosis). **(B)** DSA showing that the stenosis improved after balloon dilation (using a rapid exchange intracranial balloon dilation catheter (Neuro LPS), 1.5 mm × 12 mm). **(C)** Angiography showing occlusion of the branch vessels after NOVA stent implantation. **(D,E)** The occlusive vessels were perforated by a microguide wire, and tirofiban (0.2 mg) was injected locally using a microcatheter. **(F–H)** Angiography showing that occluded branch vessels were recanalized well.

NOVA stents were successfully implanted in all 24 cases (technical success rate 100%). The residual stenosis was less than 30% in all the patients, with no bleeding complications or mortality observed. The severity of the stenosis ranged from 75 to 95% (mean 85.9%) before treatment, which was reduced to a range from 0 to 20% (mean 8.6%) after stent placement (Figures 1, 2). The primary procedural results of all patients are summarized in Tables 2, 3. During the procedure, perforator vessel occlusion occurred in one case after stent placement caused by a “snow-plowing” effect, which was subsequently successfully recanalized using microguidewire technology and tirofiban application, showing no related clinical symptoms after the procedure (Figure 3). Symptomatic distal embolism occurred in one case, which was observed as dysarthria and decreased muscle strength after the procedure, and subsequent head MRI indicated a pontine infarction. During and after the procedure, no vessel dissection, stent displacement, HPS, or hemorrhagic transformation was observed in any patient. Among the 24 patients who experienced NOVA placement, 23 patients remained stable (*n* = 12) or showed improved symptoms (*n* = 11) at 1 week after the procedure. Only the patient who experienced the complication of distal embolism showed exacerbation of symptoms.

**Table 3:** Summary of treatment, outcome, and follow-up data for the 24 patients.

Variable	No. (%)
Technical success	24 (100%)
Residual stenosis after procedure, mean	8.6% (0–20%)
Periprocedural complications	
Perforator vessel occlusion	1 (4.2%)
Symptomatic distal embolism	1 (4.2%)
Vessel dissection	0
Hyperperfusion syndrome	0
Clinical symptom after procedure	
Stable	12 (50.0%)
Improved	11 (45.8%)
Worsened	1 (4.2%)
Follow-up	
Mean time, mean ± SD, m	12.2 ± 1.06
Imaging follow-up	
DSA	22 (91.7%)
CTA	2 (8.3%)
Significant ISR	1 (4.2%)
Minimal ISR	2 (8.3%)
Clinical follow-up	
Ischemic event	0
mRS score, mean ± SD	0.92 ± 0.83

SD, standard deviation; DSA, digital subtraction angiography; CTA, computed tomography angiography; ISR, in-stent restenosis.

### Clinical and imaging follow-up results

After discharge from the hospital, any deterioration in neurological symptoms required the patient to visit the hospital and receive a brain CT or MRI if necessary. The mean follow-up duration was 12.2 ± 1.06 months (range, 10–14 months). Clinical follow-up was performed in all patients. All patients demonstrated clinical improvement, and no symptomatic stroke events were observed during follow-up. The mean mRS score was 1.67 ± 1.09 before stenting and 0.92 ± 0.83 after stenting at the last follow-up. The patient with symptoms aggravated by distal embolism after the procedure gradually recovered his dysarthria and muscle strength through rehabilitation exercise after discharge.

Follow-up cerebral angiography was performed in 22 of the 24 patients after stenting (91.7%). Two patients refused cerebral angiography but received an CTA examination at the outpatient clinic. In both cases, there were normal flow patterns proximal to and distal to the stent, suggesting that there was no significant stenosis. Among the patients who received cerebral DSA follow-up, only one patient (4.2%) was observed to have significant ISR (55%), but with no lesion-associated symptoms. Moreover, minimal ISR (20%) was detected in two patients. These results are summarized in Tables 2, 3.

## Discussion

Implantation of a coronary DES to treat of symptomatic ICAS has been widely reported to reduce the rate of ISR and ischemic stroke recurrence, without increasing the rate of procedural complications (23–26). However, there are no reports regarding the treatment of ICAS with intracranial DES. In this report, we present our experience with the feasibility, safety, and intermediate-term follow-up after stenting with a novel intracranial DES NOVA in patients with the symptomatic severe ICAS. To our knowledge, our study is the first report on the single-center clinical experience of the NOVA stent to treat ICAS after its launch. In 2022, Miao et al. reported premarket clinical trial results of the NOVA stent, showing that compared with the Apollo stent (balloon-expandable bare-metal stent, BMS), the NOVA stent reduced the rate of ISR and stroke recurrence in patients with severe ICAS. The 1-year ISR rate in the DES group was lower than that in the BMS group (9.5% vs. 30.2%). The DES group also had a significantly lower rate of ischemic stroke recurrence (0.8% vs. 6.9%) (27). In this study, we also confirmed that intracranial DES NOVA implantation in severe ICAS is safe, feasible, and efficacious in selected patients, reducing the incidence of ISR and showing excellent midterm clinical outcomes, providing a promising option for ICAS treatment.

### Periprocedural complications

The high periprocedural complication rate is the major safety concern in intracranial stenting, with the SAMMPRIS and VISSIT trials having complication rates as high as 14.7 and 24.1%, respectively (12, 30). In the present study, we obtained a much lower periprocedural complication rate of 4.2% in a series of 24 patients treated with the NOVA stent, which is similar to the results reported in the Chinese multi-center registry study and the WEAVE trial, in which the periprocedural complication rates were 2.6 and 4.3%, respectively (15–18). Only one case experienced symptomatic distal embolism during surgery, which resulted in aggravated postoperative symptoms. The cause was considered to be fragmentation and migration of a plaque during balloon dilation. Perforator vessel occlusion occurred in one case after stent placement, caused by a “snow-plowing” effect, which was subsequently successfully recanalized, and no related clinical symptoms were presented. No vessel dissection, stent displacement, HPS, or hemorrhagic transformation occurred in any patient.

The lower periprocedural complication rate in our series cases depended on strict patient selected criteria and a rigorously controlled protocol for percutaneous transluminal angioplasty and stenting (PTAS). In our study, we performed PTAS in highly selected patients after detailed analysis of their cerebral vasculature, in which hypoperfusion was confirmed by perfusion images on responsible lesion. In fact, if hypoperfusion is the main cause of ischemic stroke, restoration of perfusion by PTAS would be helpful to prevent stroke recurrence. However, an ischemic stroke caused by thromboembolism from atherosclerotic plaques or occlusion of perforators might be best treated using medication. To reduce operation-related complications, some techniques could be considered, including application of a shuttle long sheath and an intermediate catheter to provide stable support during PTAS procedure. In addition, given the stiffer nature of the DES delivery system compared with the self-expandable stent system, the intermediate catheter is advised to be placed as close to the lesion segment as possible to obtain stable support during stent delivery. Then, to reduce the risk of vessel dissection or rupture, the diameter of the balloons and stents should be selected to a size that is slightly smaller than the diameter of the adjacent normal artery, and the stent should be deployed with very slow balloon inflation. Furthermore, to reduce the risk of hyperperfusion or cerebral hemorrhage injury, the balloon should be deflated slowly while the patient’s SBP is strictly controlled below 120 mmHg or 20 mmHg lower than the basic SBP. Benefiting from these methods, we achieved 100% technical success and a 0 % incidence of vascular injury and HPS.

### The incidence of ISR and midterm efficacy

The high ISR incidence rate is another factor that limits the application of stents in ICAS. ISR occurred in 15 to 30% of patients who received self-expanding or balloon-mounted BMS within a year (13, 14). ISR after PTAS significantly increases the rate of ischemic stoke recurrence, affecting mid-term and long-term prognosis (13). The DES is designed to reduce ISR after stent implantation by inhibiting the migration and proliferation of vascular endothelial cells and smooth muscle cells (21, 22). DESs have been promoted as first-line devices in coronary artery intervention because of their apparent inhibition of ISR (31). In some previous studies, the use of coronary DESs to treat intracranial stenosis has been reported to exhibit good feasibility and safety, and efficacious clinical outcomes (23–26). Compared with bare-metal stents, the coronary DESs used for intracranial stenosis showed lower rates of ISR and stroke recurrence (32–34). Recently, a meta-analysis of DES treatment for ICAS, the ISR rate was 4.1%, while the rate of symptomatic ISR was only 0.5%, which was similar to our result of 4.2% ISR rate without recurrent symptoms (35). Although DESs can greatly reduce ISR, they also lead to delayed stent endothelialization, resulting in stent thrombosis, even at 6 to 12 months after stent implantation (36). This seemed to be resolved by prolonging the duration of the administration of dual antiplatelet therapy. However, whether prolonging the duration of dual antiplatelet therapy increases the risk of intracranial hemorrhage was not observed in this small sample of cases during 1-year follow-up, thus larger sample size studies are needed.

### Brain tissue toxicity

The other major concern with DESs is the neurotoxicity of the drugs. Generally, the drugs used in DESs are anticancer drugs (paclitaxel or sirolimus) that are slowly released into the cerebrovascular system and affect brain tissue. The NOVA stent is a sirolimus-eluting stent system created using the electrografting technique and designed with a rapid exchangeable balloon. The density of sirolimus at the topcoat of the NOVA stent is 1.3 μg/mm^2^. No neurotoxicity was discovered in previous animal experiments before marketing, and in the subsequent randomized clinical trial, or our series cases, which indicated that the NOVA stent was safe to use within the intracranial vasculature (27).

### The NOVA stent vs. coronary DESs

NOVA DES is the world’s first balloon-expandable DES specifically designed to treat ICAS, and also is the world’s first healing-oriented intracranial stent. The NOVA stent has the following advantages over current coronary DESs: First, the material of the stent is different. The NOVA stent is made of 316 L stainless steel, while coronary DESs are mostly made of cobalt-chromium alloy. Considering that cerebral arteries are more tortuous than coronary arteries, stainless steel has lower yield strength than cobalt-chromium alloy and better adherence after stent implantation. Although cobalt-chromium alloy can make the stent beam thinner, it causes a high rate of stent thrombosis and restenosis, and post-stent expansion is usually required after implantation to ensure full adherence to the vascular wall. Second, the NOVA stent has better coating technology to promote the stent endothelialization process. It uses a unique healing-guided design-patented eG™ electron grafting coating technology, which can promote endothelial healing and reduce restenosis. Drug release and pharmacokinetic studies showed that the drug release cycle coincides with the vascular injury healing “time window” after stent placement, with the drug being completely released in 28 days, consistent with the smooth muscle cell proliferation time curve, achieving synchronous inhibition of vascular wall smooth muscle cells and reducing restenosis. The level drops below the effective concentration in 60 days, promoting endothelial healing, and it is almost completely metabolized in 90 days, eliminating the potential neurotoxic safety hazard of the drug. Tracking endothelial cell dynamics, the endothelial healing speed of eG™-coated stents is 2.5 times that of bare metal. Compared with most coronary DESs, such as the XIENCE DES (Abbott Healthcare, Chicago, IL, United States), NOVA is more effective in reducing post-stent inflammation, promoting post-stent endothelial healing, and reducing restenosis. Third, the neurotoxicity of the drug was strictly tested before marketing. Animal experimental neurotoxicity studies assessed that after NOVA intracranial drug-eluting stent placement, there were no injuries around the vessels or in brain tissues on subsequent brain MRI, no bleeding or infarction was seen in the distal brain tissue, and no degenerative changes in the brain tissue, gliosis, cell necrosis, or inflammation were observed, with no significant differences between the two groups compared with bare metal stents. However, most coronary DESs have not been tested on animals for their neurotoxicity.

## Conclusion

This study indicated a favorable midterm clinical outcome of the novel intracranial DES NOVA stent, with a low rate of ISR and without increasing the incidence of periprocedural complications in highly selected patients. The NOVA stent represents a promising new therapy to treat ICAS. However, the clinical results need to be further verified by larger sample size controlled studies with longer follow-up periods.

## Data availability statement

The original contributions presented in the study are included in the article/supplementary material, further inquiries can be directed to the corresponding author.

## Ethics statement

The studies involving humans were approved by the Ethics Committee of Tongji Hospital affiliated to Tongji University. The studies were conducted in accordance with the local legislation and institutional requirements. Written informed consent for participation was not required from the participants or the participants' legal guardians/next of kin in accordance with the national legislation and institutional requirements. Written informed consent was obtained from the individual(s) for the publication of any potentially identifiable images or data included in this article.

## Author contributions

LM: Data curation, Writing – original draft. FW: Methodology, Validation, Writing – review & editing. HF: Data curation, Formal analysis, Resources, Writing – original draft. SY: Conceptualization, Software, Writing – review & editing. J-CX: Formal analysis, Investigation, Methodology, Resources, Writing – review & editing. Y-SC: Funding acquisition, Resources, Supervision, Visualization, Writing – review & editing. CF: Funding acquisition, Resources, Writing – original draft, Writing – review & editing.
